# Dissociative states in dreams and brain chaos: implications for creative awareness

**DOI:** 10.3389/fpsyg.2015.01353

**Published:** 2015-09-07

**Authors:** Petr Bob, Olga Louchakova

**Affiliations:** ^1^Center for Neuropsychiatric Research of Traumatic Stress, Department of Psychiatry and UHSL, First Faculty of Medicine, Charles UniversityPrague, Czech Republic; ^2^Central European Institute of Technology, Faculty of Medicine, Masaryk UniversityBrno, Czech Republic; ^3^Department of Public Health Sciences, University of California, DavisDavis, CA, USA; ^4^Sofia UniversityPalo Alto, CA, USA

**Keywords:** dreams, dissociation, self-organization, chaos, creativity

## Abstract

This article reviews recent findings indicating some common brain processes during dissociative states and dreaming with the aim to outline a perspective that neural chaotic states during dreaming can be closely related to dissociative states that may manifest in dreams scenery. These data are in agreement with various clinical findings that dissociated states can be projected into the “dream scenery” in REM sleep periods and dreams may represent their specific interactions that may uncover unusual psychological potential of creativity in psychotherapy, art, and scientific discoveries.

## Introduction

A particular unexplained question is whether it may be possible for an individual to have a “transcendental experience” in which consciousness transcends its own symbolic self-understanding and abstract thinking and how these experiences may be related to particular aspects of consciousness and their complex organization in the brain. Recent findings indicate that conscious states can be described as representations of brain states and related to dynamics of large-scale neuronal networks (Freeman, 1991, 1999, 2000; Singer, 2001; Varela et al., 2001; Rees et al., 2002; Fingelkurts et al., 2010, 2013; Bob, 2011; Fingelkurts and Fingelkurts, 2012).

According to some recent theoretical viewpoints subjective experience can be represented in the brain by a “cerebral mental field” which refers to the mind as a system property related to the synchronized activities of large numbers of neurons (Libet, 2006). These specific complex processes linking the brain and its cognitive “mental field” may help to describe relationships between subjective experience and brain activation patterns on various functional levels of organization (Freeman, 1991, 1999, 2000; Melancon and Joanette, 2000; Varela et al., 2001; Korn and Faure, 2003). In addition, these findings show that not only brain generates consciousness, but also consciousness affects the brain. This complex organization of subjective mental experiences plays a role in spatio-temporal activities of the brain, which via various feedback loops are associated with the spatial-temporal organization of the mind processes (Freeman, 1991, 1999, 2000; Fingelkurts et al., 2010, 2013; Bob, 2011; Fingelkurts and Fingelkurts, 2012; Bass, 2014; Tang et al., 2015).

In this context, recent findings indicate that distributed patterns of neuronal activities are specifically linked within neural assemblies that tend to create coherent systems, which enable mental representations related to perceptual and cognitive functions, memory processes, and conscious awareness (Freeman, 1991; Singer, 2001; Rees et al., 2002). In addition, according to the recent evidence these neural mechanisms that enable conscious integration are significantly affected by various stressful and traumatic experiences that may determine dissociation of conscious awareness and memory (Li and Spiegel, 1992; Putnam, 1997; Bob, 2003; Spiegel, 2012).

## Dissociative States in Dreams

Dissociative states usually emerge as a disconnection and switch between different mental states due to a disconnection between memories related to traumatic or stressful experiences that disturb conscious awareness and experience of the self (Li and Spiegel, 1992; Putnam, 1997; Bob, 2003; Spiegel, 2012). In this context, dissociation can be defined as a partial or total disconnection between memories of the past, awareness of identity and of immediate sensations, and control of bodily movements often resulting from traumatic experiences, intolerable problems, or disturbed relationships (Li and Spiegel, 1992; Bob, 2003; Colman, 2003). Due to this disconnection of memory and identity dissociation is related to disability to integrate some mental contents into conscious awareness (Bernstein and Putnam, 1986). In agreement with new DSM-V criteria, dissociation may be defined: “as a disruption of and/or discontinuity in the normal, subjective integration of one or more aspects of psychological functioning, including—but not limited to—memory, identity, consciousness, perception, and motor control” (Spiegel, 2012; American Psychiatric Association, 2014).

Dissociative mental states also manifest during dreams and may be typically related to discontinuities and shifts in dream scenes, which according to some psychological studies of dreams may specifically manifest in pathological processes related to nightmares and recurrent dreams linked to traumatic experiences (Ferenczi, 1934; Levitan, 1980; Hartmann, 1998; Bob, 2004; Schonhammer, 2005). Intriguing relationships between dreams and the dissociative states were also reported in cases of the so-called “alter personalities” in multiple personality disorder (MPD), which may manifest on parallel levels in dreams and during personality alterations in hypnosis (Jeans, 1976; Marmer, 1980a,b; Salley, 1988; Putnam, 1989; Brenner, 1996, 1999, 2001; Bob, 2004). For example, Barrett (1994, 1995, 1996) reported some cases of MPD in which alter personalities manifested in dream scenes. These case studies suggest that dream “personalities” may manifest as hallucinated projections of various aspects of the fragmented self that may be understood as prototypes of these alter personalities (Bowers and Brecher, 1955; Watkins and Watkins, 1979–1980; Merskey, 1992; Rickeport, 1992; Watkins, 1993; Lynn et al., 1994; Barrett, 1995; Bob, 2004). In this context, Bowers and Brecher (1955) reported that fragmented aspects of the self, very similar to altered personalities in MPD, may manifest during hypnosis also in individuals without multiple personality structures and also they may be observed in dreams and associations using Rorschach test. Taken together these reported studies suggest that dissociative mental states may be projected as specific types of hallucinatory experiences during dreams and may be identified as parts of dream scenes (Gabel, 1989; Hartmann, 1998; Bob, 2004). An important implication of these data is that dreaming processes may represent a conscious reflection of dissociative states represented during memory processing in REM sleep (Gabel, 1989; Rotenberg, 1992; Bob, 2004; Eisser, 2005; Stickgold and Walker, 2005).

## Dreams and Brain Chaos

Recent findings indicate that some modalities of awareness and attention used for self-monitoring and for the internal explorations of one’s consciousness such as dreams, meditation and various forms of creative awareness may be at certain levels of brain functions understood using theories of non-linear dynamics, chaos and self-organization (Freeman, 1991, 1999, 2000; Elbert et al., 1994; Melancon and Joanette, 2000; Bob, 2003, 2011; Korn and Faure, 2003; Fingelkurts et al., 2013).

In the scientific history, the chaotic processes were for the first time documented in the last years of 19th century in the work by Poincaré (1908/1998). In his “Science and method” Poincaré (1908/1998, p. 68) wrote: “A very small, unnoticeable cause can determine a visible very large effect; in this case we claim that this effect is a product of random …”.

In the dynamic state of chaos very small changes in a system can result to very large differences in the system’s behavior (the so-called butterfly effect, which metaphorically means that the flapping of a butterfly’s wings in one part of the world later may cause a tornado in a distant part of the world). Chaos represents a dynamic pattern of activity that occurs when a system involves a large number of interlinked and simultaneously active states, which can lead to self-organization (Freeman, 1991, 2000; Elbert et al., 1994; Korn and Faure, 2003; Bob, 2015).

Seminal contributions to this field of research reported Freeman (Freeman, 1991, 1999, 2000; Elbert et al., 1994; Bob, 2011), who was particularly interested to explore how brain generates cognitive processing, intentionality, and meaning. In his research Freeman found that brain activities may manifest chaotic behavior. Freeman also proposed that chaos could underlie basic forms of collective neural activity in perceptual processing including ability to access memorized sensory patterns and learning of novel sensory information. In cognitive processes chaos may explain brain ability to respond flexibly to the outside world and to generate novel activity patterns that are subjectively experienced as “novel” ideas that enable complex dynamic behavior of the brain and intentional behavior (Freeman, 1991, 1999, 2000).

Recent findings indicate that deterministic mechanisms of brain activity as well as chaotic neural patterns are important for brain functioning (Freeman, 1991, 1999, 2000; Elbert et al., 1994; Birbaumer et al., 1995; Kantz and Schreiber, 1997; Bob, 2011). In this context, mental states and brain activities related to intentional goals are predictable and deterministic. On the other hand brain processes related to great sensitivity to very small changes, mainly in initial stages of a neural processes, seem to be characterized by chaotic neural activities and mental states, for example during free floating divergent thinking or search related mental events (Freeman, 1991, 1999, 2000; Elbert et al., 1994; Globus and Arpaia, 1994; Birbaumer et al., 1995; Faure and Korn, 2001; Meyer-Lindenberg et al., 2002; Bob, 2011; Yoruk and Runco, 2014). These chaotic brain activities with increased sensitivity to “initial conditions” underlying novel trends in brain processes are related to a very large number of interacting and interlinked neural states that are sensitive and unstable because of a competition of many neural patterns (Freeman, 2000; Korn and Faure, 2003). According to these findings chaos may enable flexible brain responses to some external stimuli associated with novel neural activities, behavior and cognitive processing, for example experiences of original ideas and creativity (Skarda and Freeman, 1987; Freeman, 1991, 2000, 2001; Elbert et al., 1994; Melancon and Joanette, 2000; Korn and Faure, 2003; Bob, 2011). Chaotic states in the brain may manifest in various cognitive processes as for example in dreams (Kahn and Hobson, 1993; Kahn et al., 2000, 2002; Bob, 2011; Kahn, 2013), dissociative states (Pediaditakis, 1992; Putnam, 1997; Sel, 1997; Bob, 2003) and may characterize specific processes in development of mental disorders, as for example in depression or schizophrenia (Gottschalk et al., 1995; Huber et al., 1999; Paulus and Braff, 2003; Bob, 2011).

Chaotic brain states are usually related to activities in various independent regions that process information in parallel distributed mode (Tirsch et al., 2004). This parallel distributed processing may manifest in transient states and fluctuations of increased or decreased complexity that in cases of low associated strength among the parallel distributed information subsystems may lead to dissociated mental states (Mc Clelland et al., 1986; Li and Spiegel, 1992; Butler et al., 1996; Bob, 2003). Due to these decreased levels of association between some information processes, dissociation manifests as disturbed continuity between interacting mental states (Li and Spiegel, 1992; Bob, 2003).

Dream scenes mainly occur in the REM sleep and include mental images, thoughts, sounds, and other sensory experiences in various sequences that may be episodically disturbed by discontinuous shifts in dream narratives (Kahn and Hobson, 1993; Faw, 1997; Kahn et al., 1997, 2000, 2002; Stickgold et al., 2001; Hobson and Pace-Schott, 2002; Kahn, 2013). According to some findings neural correlates of these discontinuous shifts in a dream scenery are rapid shifts in neural patterns related to self-organized neural activities, mainly associated with activities of cholinergic pontogeniculoocipital (PGO) systems (Hobson and McCarley, 1977; Quattrochi et al., 1989; Hobson, 1990; Kahn and Hobson, 1993; Stickgold et al., 1994, 2001; Kahn et al., 1997, 2000, 2002; Kahn, 2013). In addition, there is also evidence that the PGO activity is correlated with increased firings in the visual cortex and lateral geniculate bodies participating in formation of images (Callaway et al., 1987; Singer, 1989; Kahn and Hobson, 1993; Porte and Hobson, 1996; Stickgold et al., 2001; Kahn, 2013). During these self-organizing processes brain responses may become very sensitive with respect to very small stimuli which may lead to chaotic bifurcations that cause rapid shifts in patterns of neural activities related to increased cholinergic and decreased aminergic input onto forebrain structures (Kahn and Hobson, 1993; Stickgold et al., 1994; Kahn et al., 2000, 2002; Kahn, 2013). According to recent findings these chaotic processes may be characterized by significant transitions of dream objects and sceneries due to a competition and interference of various dream images, which lead to dream discontinuities and multiple transitions of neural firing patterns (Tender and Kramer, 1971; Kaczmarek and Babloyantz, 1977; Elazar and Hobson, 1985; Rotenberg, 1992; Kahn and Hobson, 1993; Kahn et al., 1997, 2000, 2002; Kahn, 2013).

## Dreams, Conscious Awareness, and Novel Ideas

Reported findings suggest that dreams may reflect dissociative mental states that especially manifest as discontinuous jumps in the dream scenery (Salley, 1988; Gabel, 1989; Bob, 2004), which in a similar way as dissociative states during waking may be related to chaos and self-organization in the brain (Pediaditakis, 1992; Putnam, 1997; Sel, 1997; Bob, 2003, 2011). These findings are in accordance with experiences and case studies in psychotherapy, which indicate that dreams are not random processes and may be meaningful for self-discovery and personal growth (Jeans, 1976; Marmer, 1980a,b; Salley, 1988; Barrett, 1994, 1995, 1996; Brenner, 1996, 1999, 2001; Bob, 2004).

A specific feature of chaotic neural states is that they can generate novel patterns of neural activities and novel synaptic connections, which may link less associated or dissociative mental states into a coherent whole (Kahn and Hobson, 1993; Kahn et al., 2000). For example, several findings suggest that high complexity and self-organization manifest also during creative divergent thinking (Combs, 1996; Mölle et al., 1996). Specifically in the REM sleep these integrative processes support “binding” functions of dreams, which connect dream images coherently together that enables novel creative unity of conscious experience (Revonsuo and Tarkko, 2002). These findings suggest that dreams may reflect new integrations of dissociated mental states related to traumatic and stressful experiences and may also increase creative potentialities in various cases of artistic inventions, scientific discoveries and deep insights related to meta-cognitive, transcendental or religious experiences reflecting certain forms of “preconscious” or “unconscious” intelligence (Haule, 1984; Barrett, 1993; Strunz, 1993; Baylor, 2001; Schaverien, 2005; Louchakova, 2006; Edwards et al., 2013; Dresler et al., 2015).

## Conclusion

Chaos and self-organization manifest in brain and cognitive functions and may be specifically related to dissociative states characterized by rapid shifts between disconnected mental states that in the case of dreams are likely specifically related to PGO bursts of neural activity (Kahn and Hobson, 1993; Putnam, 1997; Bob, 2003, 2011; Korn and Faure, 2003; Kahn, 2013).

Although the current literature and evidence about these links between dreams and dissociated states are limited, they provide useful explanatory scheme for future research that could explain more detailed connections of self-organizing theory of dreams and dissociative states. For example, Tart (2009) suggests that hypnotic techniques provide a useful framework for experimental studies of dreams and other clinical data show that also dreams in patients who experienced serious traumatic events could open new perspectives in this research (Bob, 2004; Rotenberg, 2014). These studies may also provide new theoretical framework for understanding of connections between dreams and brain functions related to unusual creative potential of unconscious mental processing as well as important findings for psychotherapy that using “dream analysis” may open novel insights and creativity in individual life and help to resolve traumatic experiences linked to various mental disorders.

Taken together these data suggest a perspective for further research that dissociated mental states may be projected into a dream scenery during REM sleep and dreams may represent specific interactions of dissociated contents, which may create the content of the dream (Salley, 1988; Gabel, 1989; Bob, 2004). These data also suggest that neural chaotic states during dreaming may represent underlying neural processes that enable new integration of dissociated contents of memory which may generate novel ideas, insights and other creative conscious states.

## Conflict of Interest Statement

The authors declare that the research was conducted in the absence of any commercial or financial relationships that could be construed as a potential conflict of interest.
